# The influence of beam delivery uncertainty on dose uniformity and penumbra for pencil beam scanning in carbon-ion radiotherapy

**DOI:** 10.1371/journal.pone.0249452

**Published:** 2021-04-01

**Authors:** Yue Li, Yunzhe Gao, Xinguo Liu, Jian Shi, Jiawen Xia, Jiancheng Yang, Lijun Mao

**Affiliations:** 1 Institute of Modern Physics, Chinese Academy of Sciences, Lanzhou, China; 2 University of Chinese Academy of Sciences, Beijing, China; 3 Huizhou Research Center of Ion Science, Huizhou, China; St. Vincent Medical Center, UNITED STATES

## Abstract

The dose uniformity and penumbra in the treatment field are important factors in radiotherapy, which affects the outcomes of radiotherapy. In this study, the integrated depth-dose-distributions (IDDDs) of 190 MeV/u and 260 MeV/u carbon beams in the active spot-scanning delivery system were measured and calculated by FLUKA Monte Carlo simulation based on the Heavy Ion Medical Machine (HIMM). Considering the dose distributions caused by secondary particles and scattering, we also used different types of pencil beam (PB) models to fit and compare the spatial distributions of PB. We superposed a bunch of PB to form a 20×20 cm^2^ treatment field with the double Gaussian and double Gaussian logistic beam models and calculated the influence of beam delivery error on the field flatness and penumbra, respectively. The simulated IDDDs showed good agreement with the measured values. The triple Gaussian and double Gaussian logistic beam models have good fitness to the simulated dose distributions. There are different influences on dose uniformity and penumbra resulting from beam uncertainties. These results would be helpful for understanding carbon ion therapy, and physical therapists are more familiar with beam characteristics for active scanning therapy, which provides a reference for commissioning and optimization of treatment plans in radiotherapy.

## 1. Introduction

Carbon ion radiotherapy (CIRT) is an attractive cancer treatment modality, especially for the treatment of hypoxic tumors. Recently, the treatment of tumors with CIRT has garnered tremendous attention because of their high dose localization in the Bragg-peak region [1].

At present, there are two main types of carbon ion beam delivery systems, both of which have been applied in clinical trials. One is the passive beam delivery system represented by the Heavy-Ion Medical Accelerator in Chiba (HIMAC) treatment device from Japan [2], the other is the active beam delivery system represented by Gesellschaft für Schwerionenforschung (GSI) from Germany [3]. The passive delivery system uses additional hardware to configure the beam, while an active delivery system directs the pencil beam to the tumor area through active control of an accelerator or other device. An important drawback of the passive uniform scanning technique is the passive elements (e.g., range shifter) in the nozzle, which increases the lateral penumbra in the patient [4]. The active spot scanning technique can theoretically improve the dose conformation [5].

Currently, pencil beam (PB) and Monte Carlo (MC) algorithms are two types of dose calculation algorithms, which are used in the treatment planning system (TPS) [6]. MC methods play key roles in the scientific research and dose verification of radiation therapy and nuclear medical imaging by accurately simulating the interaction between various particles and atoms in substances [7]. Many MC procedures are gradually applied to CIRT. Although the Monte Carlo method is limited to replace the analytical algorithm because of the long computing time, basic research on the MC method as the core algorithm of the TPS has become a hot topic in scientific research. In particular, the MC method can save expensive experimental beamtime [7].

There are already some MC simulation works for the treatment nozzle. However, these simulations for the treatment nozzle are mainly focused on proton radiotherapy, and there are comparatively fewer studies on the treatment nozzle of CIRT using other MC codes [8–11]. Nevertheless, most of these simulations mainly focused on passive CIRT. More importantly, the secondary neutron dose, a harmful secondary particle, can be significantly decreased by using an active scanned beam in CIRT, which has been demonstrated experimentally and calculated through MC simulation [12]. Therefore, active spot scanning is a promising direction in the development of CIRT. Although active spot-scanning technique is superior to passively scattered or uniform scanning techniques in dosimetry, numerous limitations exist in the present active spot-scanning methods. In particular, compared to passively scattered radiotherapy, active spot scanning is highly vulnerable to various uncertainties. There is still much effort to investigate the active scanning method, including treatment plan dosimetry.

We chose FLUKA to model our beamline because it has been successfully used to simulate scanning ion beamlines [7, 13–16], especially for medical applications. Therefore, the active scanning treatment nozzle of HIMM was constructed with the FLUKA simulation tool for the first time in the present study, and was used to study the change in the depth-dose curves of carbon ions with different energies. In MC simulation, to increase the accuracy of the simulation, it is usually achieved by increasing the number of incident primaries. However, this manner will largely increase the commissioning time. Thus, the main problem of dose calculation is the contradiction between calculation accuracy and calculation speed. According to the actual needs, software with fast execution speed is needed to complete the simulation calculation, and then the result processing is carried out with the software with convenient graphics processing. Programing platforms such as MATLAB make it easier to achieve [17].

The low-dose envelope refers to the low dose distribution around the transverse beam, which is located far away from the axis and is mainly composed of secondary particles. In many primary versions of TPSs, a single Gaussian model is often used to calculate the transverse dose distribution in water [18–20], including ciPlan developed by the Institute of Modern Physics (IMP). However, the single Gaussian model does not sufficiently consider the low-dose envelope of the pencil beam, which leads to inaccurate dose calculation. Therefore, there have been many attempts to approximately include this low dose envelope in PB algorithms in recent years. Many accurate pencil beam models have been proposed, such as the double Gaussian model and triple Gaussian model. Recently, a double Gaussian-logistic model proposed by Zhang *et al*. has a better fitness than the triple Gaussian model [21]. Although the commercially offered MC algorithms are very efficient compared to generic research-based codes, their dose calculation and plan optimization speeds are still slower than pencil beam (PB) algorithms. To include the low dose envelope as large as possible and decrease the dose calculation time, we analyzed the total three-dimensional dose distribution in the water tank obtained by MC simulation and acquired the input parameters for the PB dose distribution using MATLAB software.

The importance of accurate treatment plan dosimetry in radiation therapy cannot be overstated. Uncertainties of beam delivery systems always occur in scanning beam systems, including spot size, spot position, and ion number per spot. These delivery uncertainties were introduced into our simulations as Gaussian-like distribution random offsets. To balance the accuracy of treatment and treatment time, to our knowledge, we first propose to use the MC simulated dose distribution to fit and select the best pencil beam model, and then analyze the influence of the uncertainty of the beam delivery system on the dose uniformity and penumbra in the radiation field.

## 2. Materials and methods

### 2.1 Measurement and calculation of depth dose distributions of 190 MeV/u and 260 MeV/u carbon beam

The accuracy of the MC method is in good agreement with the experimental data of a particle transport [22]. To clarify the reliability of the FLUKA simulation, we first measured the integrated depth-dose-distributions (IDDDs) according to the method described by Mirandola *et al*. [23]. Briefly, the water tank measurement system, PTW MP3-P water tank (Freiburg, Germany), was placed on the isocenter position of the treatment couch, and the doses were measured with a parallel plate ionization chamber (PTW Bragg Peak Ionization Chamber T34070 (Freiburg, Germany). The wall of the tank was PMMA. The carbon beam enters the tank in the horizontal direction through the incident window. Eventually, the depth-dose curves were acquired using a dosimeter (PTW Tandem Dual Channel Electrometer) and displayed by the corresponding software system. The simulation and measured results were normalized at the entrance of the water tank.

### 2.2 HIMM treatment nozzle description and settings in FLUKA

Although the ionization chamber can improve the measurement resolution to a certain extent, it is time-consuming and cannot accurately measure the dose below the mGy level [24]. Fortunately, the dose contribution of the low-dose envelope can be obtained by the MC simulation. This method is no longer limited by the experimental conditions and the accuracy of the measurement equipment. It is often referred to as the "gold standard" in modern radiation therapy physics. Therefore, this study uses the FLUKA simulation platform to simulate the carbon ion PB, and analyzes its dose distribution in water.

For the physical set-up, the DEFAULTS card was set to the HADROTHE option in the FLUKA input file, which is recommended for CIRT [25]. The cutoff energy of particle transport was set at 10^−1^ MeV, except for neutrons and (anti) neutrons. In this work, 10^6^ incident particles were counted in each simulation. The grid is divided into 0.5 mm per bin in *x* and *y* directions and 1 mm per bin in *z* direction. An ideal PB model was used, and the cross-section was a Gaussian distribution in the lateral direction.

**Fig 1:**
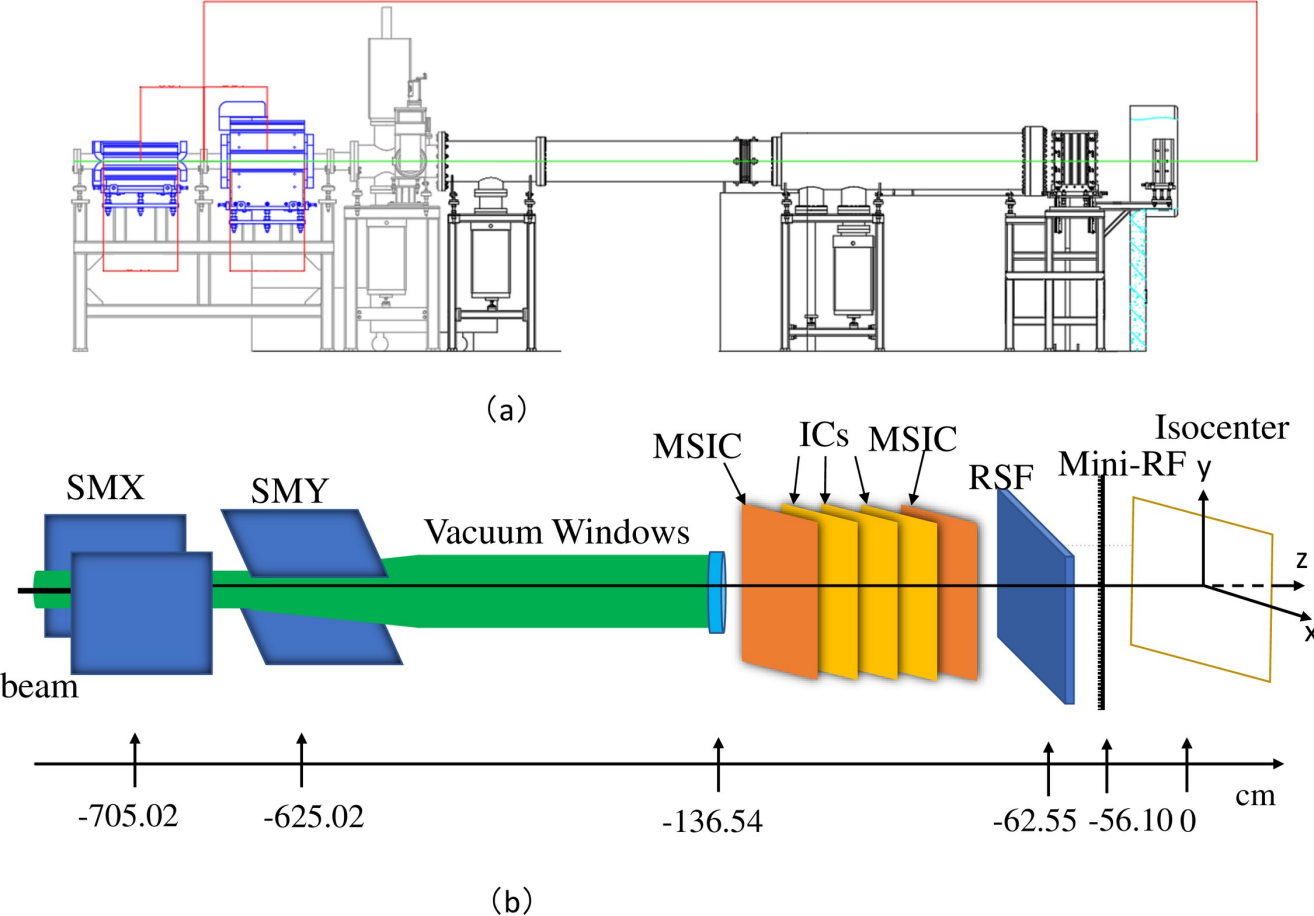
Fixed horizontal beamline for modulated spot scanning delivery. (a) Layout of the HIMM; (b) The PB scanning treatment head implemented in the MC code, showing the incident beam direction, scanning magnets (SMX, SMY), ionization chambers (IC); mini-RF, ripple filter; RS, range shifter. Unit: cm.

Based on FLUKA MC, an accurate HIMM treatment nozzle model was established to simulate the radiation field after the beam passed through the treatment terminal to accurately simulate the dose deposition of the beam during the treatment. In terms of geometry, different approximations were adopted to describe the composition of the HIMM nozzle (Fig 1). The carbon ion beam generated and accelerated by the HIMM ion source, cyclotron, and synchrotron in the energy range of 100–400 MeV/u is PB, which needs to be widened by a ripple filter in order to broaden the pristine Bragg peaks and reduce treatment time [7].

The magnetic center of the longitudinal scanning magnet is located at Z = −700 cm, and the magnetic center of the horizontal scanning magnet is located at Z = −630 cm. The vacuum chamber is the path of the carbon ion beam in the treatment nozzle, which is evacuated to reduce scattering. The total length of the vacuum chamber was 337 cm, and there were very thin aluminum windows on both sides. The dose monitors are three ionization chambers (ICs) to meet the redundant measurement required by the regulations of the Class III medical device. The three ICs are filled with dry gas, and the two sides are thin polyimide windows, located ~ 300 cm away from the isocenter. For the MC dose calculations, the thickness was fine-tuned according to the dose measurement [7]. A ripple filter was used to widen the pristine Bragg peak to form a mini-Spread Out Bragg Peak (SOBP) with a 4 mm full width at half maximum (FWHM), which reduces the number of layers of the tumor target during conformal irradiation. The treatment nozzle is also equipped with range shifters to treat superficial tumors. The material of the range shifter is polyethylene, 62.55 cm away from the isocenter plane. The primary and secondary dose monitoring ionization chamber has the same performance, which is primarily composed of an incident and exit window, a central anode, and two cathodes. As a beam position detector, the striped ionization chamber is composed of an incident and exit window, a central cathode, and two X-and Y-direction position poles.

### 2.3 Beam models

Different PB models, the single Gaussian model (Eq 1), double Gaussian model (Eq 2), triple Gaussian model (Eq 3), and double Gaussian logistic model (Eq 4) were used to fit the 3D dose distribution data in water obtained from the FLUKA simulations. The starting values for all models are listed in Table 1. The single Gaussian model is used to fit the FLUKA measurement curve, and the parameter *σ* obtained by fitting was used for the initial value of other models.

**Table 1:** The initial values for all models.

Parameters	double Gaussian model	triple Gaussian model	double Gaussian-logistic model
*f*_1_	0.995	0.8	0.8
*f*_2_	-	0.1	0.1
*σ*_1_	0.7*σ*	0.6*σ*	0.6*σ*
*σ*_2_	*σ*	2*σ*	2*σ*
*σ*_3_	-	6*σ*	-
s	-	-	3*σ*

Note: The dashed symbols in the table indicate no parameters used in this model.

$$L_{k}(x,y,x^{\prime },y^{\prime },Z(x^{\prime },y^{\prime },z))=\frac{1}{{2\pi \sigma (z)}^{2}}e^{-\frac{{\left(x-x^{\prime }\right)}^{2}+{\left(y-y^{\prime }\right)}^{2}}{2{\sigma (z)}^{2}}}$$ (1)

$$L_{k}(x,y,x^{\prime },y^{\prime },Z(x^{\prime },y^{\prime },z))=\frac{f_{1}(Z)}{{2\pi \sigma_{1}(z)}^{2}}e^{-\frac{{\left(x-x^{\prime }\right)}^{2}+{\left(y-y^{\prime }\right)}^{2}}{2{\sigma_{1}(z)}^{2}}}+\frac{1-f_{1}(Z)}{{2\pi \sigma_{2}(z)}^{2}}e^{-\frac{{\left(x-x^{\prime }\right)}^{2}+{\left(y-y^{\prime }\right)}^{2}}{2{\sigma_{2}(z)}^{2}}}$$ (2)

$$L_{k}(x,y,x^{\prime },y^{\prime },Z(x^{\prime },y^{\prime },z))=\frac{f_{1}(Z)}{{2\pi \sigma_{1}(z)}^{2}}e^{-\frac{{\left(x-x^{\prime }\right)}^{2}+{\left(y-y^{\prime }\right)}^{2}}{2{\sigma_{1}(z)}^{2}}}+\frac{f_{2}(Z)}{{2\pi \sigma_{2}(z)}^{2}}e^{-\frac{{\left(x-x^{\prime }\right)}^{2}+{\left(y-y^{\prime }\right)}^{2}}{2{\sigma_{2}(z)}^{2}}}+\frac{1-f_{1}(Z)-f_{2}(Z)}{{2\pi \sigma_{3}(z)}^{2}}e^{-\frac{{\left(x-x^{\prime }\right)}^{2}+{\left(y-y^{\prime }\right)}^{2}}{2{\sigma_{3}(z)}^{2}}}$$ (3)

$$L_{k}(x,y,x^{\prime },y^{\prime },Z)=\frac{f_{1}(Z)}{{2\pi \sigma_{1}(z)}^{2}}e^{-\frac{{\left(x-x^{\prime }\right)}^{2}+{\left(y-y^{\prime }\right)}^{2}}{2{\sigma_{1}(z)}^{2}}}+\frac{f_{2}(Z)}{2\pi \sigma_{2}{\left(Z\right)}^{2}}e^{-\frac{{\left(x-x^{\prime }\right)}^{2}+{\left(y-y^{\prime }\right)}^{2}}{2{\sigma_{2}(z)}^{2}}}+\frac{(1-f_{1}(Z)-f_{2}(Z))e^{-(\frac{x-x^{\prime }+y-y^{\prime }}{s(Z)})}}{s{\left(Z\right)}^{2}{\left(1+e^{-\frac{x-x^{\prime }}{s(Z)}}\right)}^{2}{\left(1+e^{-\frac{y-y^{\prime }}{s(Z)}}\right)}^{2}}$$ (4)

The specific meaning of the parameters for σ(z), s(z), and *f* in the formula has been explained in reference [21].

### 2.4 Conformation of square field

Considering the delivery uncertainty or not considering the delivery uncertainty, a 20 × 20 cm^2^ field under different conditions was simulated, and the uniformity of the square field was analyzed. Normally, the spot spacing of beam is 2–4 mm on a regular grid [26]. We assume that the scanned carbon-ion beam spots have the same lateral and longitudinal sizes in all simulations. We used the MATLAB program to investigate the effect of spot spacing on dose flatness and penumbra under ideal conditions, that is, no delivery errors of the system. All MATLAB codes are available in the S1 File.

The homogeneity of the delivered field is an important parameter in radiotherapy. In this study, dose flatness (Eq 5), a common parameter used in the field of radiotherapy, was employed to measure it [27, 28].

$$Flatness=\left(|\frac{D_{max}-D_{min}}{D_{max}+D_{min}}|\right)\times 100\%$$ (5)

Where, *D*_*max*_ is the maximum dose and *D*_*min*_ is minimum dose, respectively.

**Fig 2:**
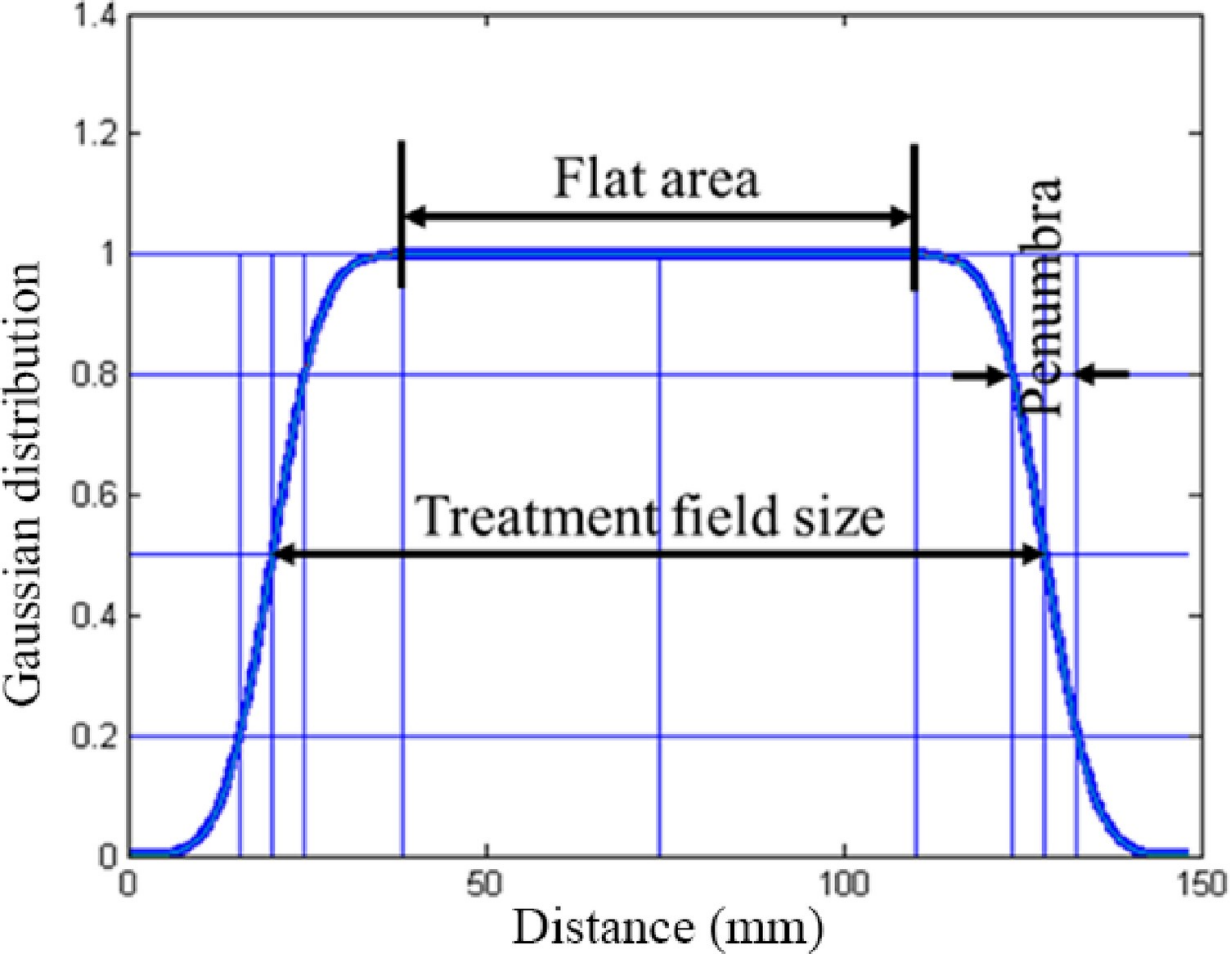
Schematic illustrations of concepts underlying the computational method.

Compared with the uniform dose in the radiation field center region, the dose at the edge of the field changes sharply and drops rapidly, forming the penumbra area. The width between 80% and 20% falls off, indicating the size of the penumbra (Fig 2).

For active spot-scanning beam delivery systems, the 3D dose distribution can be regarded as the superposition of the radiation dose of each spot beam. In order to calculate the flatness of dose distribution and penumbra, we used a numerical Gaussian model.

### 2.5 Variation of spot position

The accuracy of the spot position and dose delivered per spot is helpful to improve the accuracy of the beam delivery system [29].

Random error is a common phenomenon in radiotherapy, even in the testing process [5, 30]. Beam point loss and beam line loss are fewer common phenomena in reality, which are the worst-case scenario we assumed. To examine these delivery uncertainties, we used *the rand* function and *the unidrnd* function to realize spot position error (the standard deviation and loss point, respectively). For the loss point and loss line (the loss points in a row), the simulation was repeated twenty times using *the unidrnd* function. The mean flatness and penumbra were calculated. For the sake of simplicity, each irradiation point in the same energy layer is set at the same radiation dose.

### 2.6 Data analysis

The data calculated by the MC code were analyzed and plotted using Origin software (Origin 2020b version, USA). For the distribution of carbon ions, Gaussian function fitting was performed through sequential fitting Apps, and the FWHM of the spot size was acquired by Eq (6).

$$FWHM=2\sqrt{2ln2}\times \sigma \approx 2.355\times \sigma$$ (6)

For the flatness and penumbra calculation, the treatment field was formed using MATLAB software (version 2018a). The dose flatness should be less than 3% to meet the requirements of clinical treatment of the carbon ion beam.

## 3. Results

### 3.1 Depth-dose distributions and lateral-dose profiles of carbon ions

**Fig 3:**
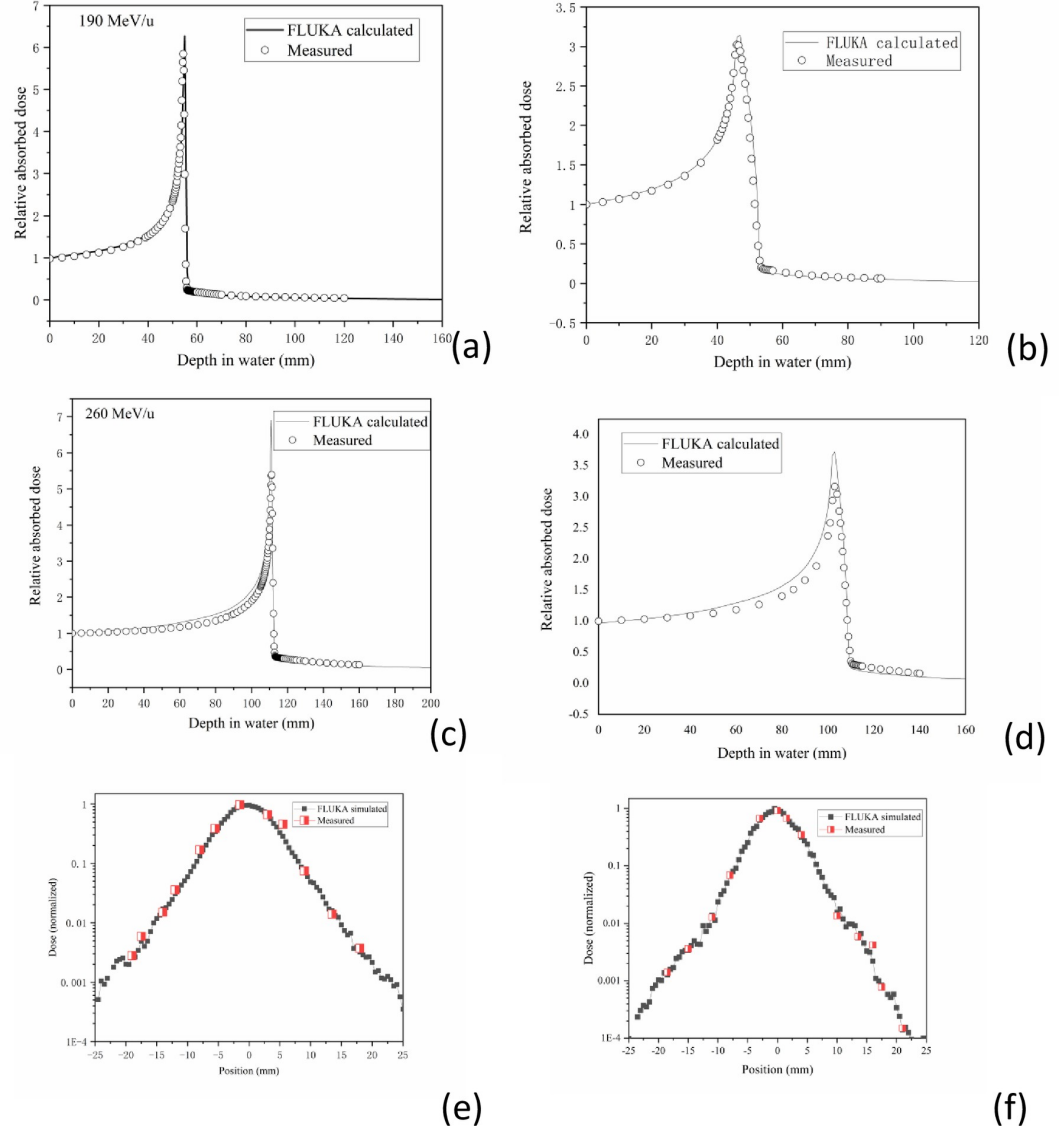
Depth-dose distributions and lateral-dose profiles of carbon ions. MC calculations of laterally IDDDs in water (solid line) in comparison to measured data (open circles) for carbon ions at 190 MeV/u ((a) w/o ripple filter, (b) with ripple filter) and 260 MeV/u carbon ion ((c) w/o ripple filter, (d) with ripple filter). MC simulations of lateral-dose profiles of carbon ions in water, with 190 MeV/u (e) and 260 MeV/u (f) energy, respectively, sampled in before the Bragg peak, in comparison to experimental measurements taken at HIMM.

The IDDDs for the representative clinical-related energy available for 190 and 260 MeV/u carbon ions were acquired with or without ripple filters (Fig 3(A)–3(D)). A good agreement between the measured and simulated IDDDs can be observed at 190 MeV/u and 260 MeV/u carbon ion beams with or without the ripple filter. The measured IDDDs were compared to MC simulations. Compared with the experimental results, the average error of the simulation results is within ± 1.5%-2.9% in the plateau region. The average error of the Bragg peak region is ± 4.2–5.6%. In addition, MC simulations of lateral-dose profiles of carbon ions in water, with 190 and 260 MeV/u energy, respectively, sampled in before the Bragg peak, in comparison to experimental measurements was done at HIMM (Fig 3(E) and 3(F). Considering unavoidable uncertainties of the measured data in the low-dose region, the agreement is quite satisfactory [25]. Therefore, FLUKA is a reliable tool to simulate the dose distribution of HIMM [31].

### 3.2 Beam models

#### 3.2.1 Carbon-ion PB distribution was modeled by Origin software

**Fig 4:**
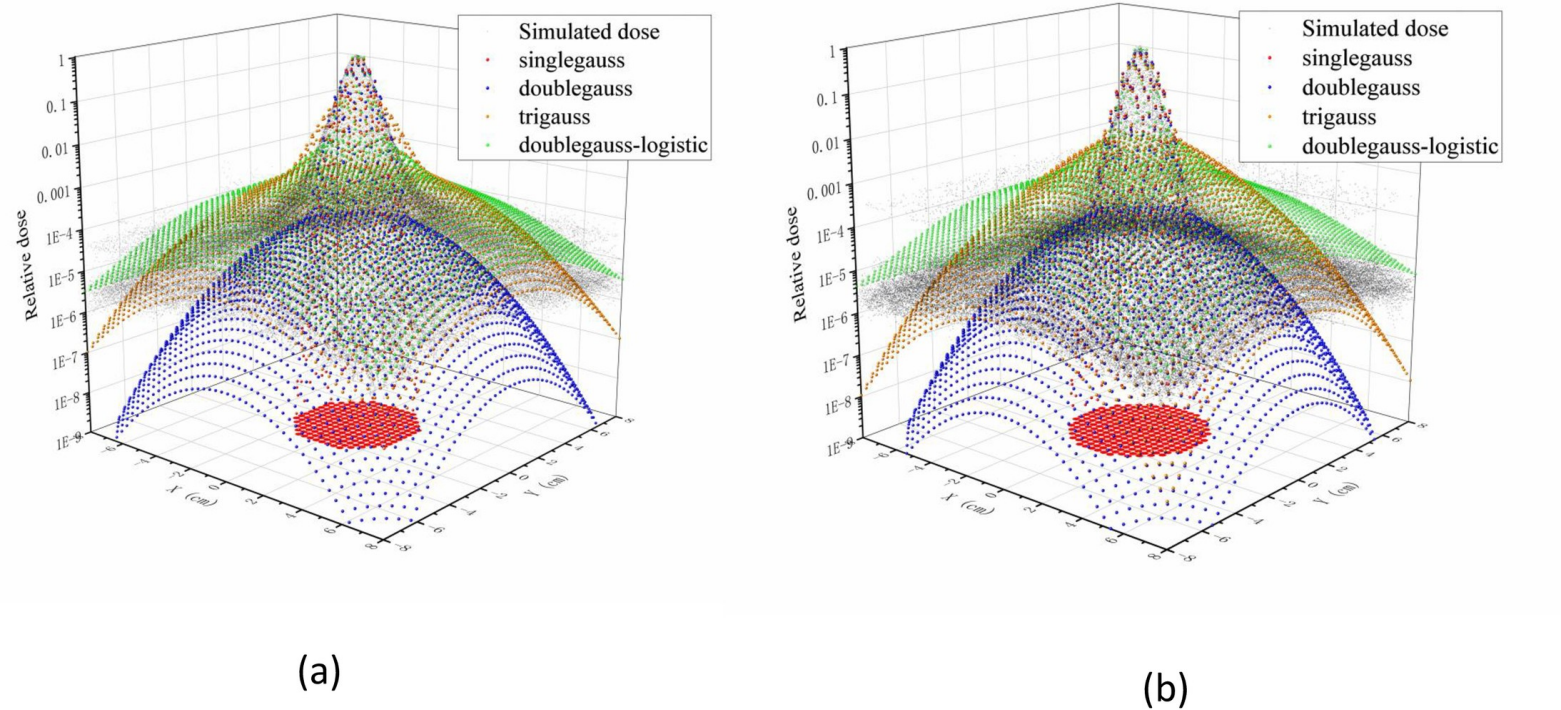
Carbon-ion PB distribution was modeled by Origin software. 3D dose distributions of the total dose for (a)190 MeV/u and (b) 260 MeV/u carbon-ion PB. All data were normalized with the maximum dose value.

To provide parameters for the following MATLAB simulation, the single Gaussian model, double Gaussian model, triple Gaussian model, and double Gaussian logistic model was used to model the physical dose distributions of 190 MeV/u and 260 MeV/u carbon beams in water using Origin software (Fig 4(A) and 4(B)). The single Gaussian width, *σ*, was 4.2 mm for 190 MeV/u. According to the starting value, we obtained the *f*_1_, *f*_2_, *σ*_1_, *σ*_2_ and *σ*_3_ parameter values. When *f*_1_, *f*_2_, *σ*_1_, and *σ*_2_ were 0.3, 0.6, 2.52, and 8.4 for the triple Gaussian model and double Gaussian-logistic model. The results of the triple Gaussian model and double Gaussian logistic model are closer to the simulated values. The accuracy of the double Gaussian-logistic model is better than that of the triple Gaussian model for the lateral distance from the axis. These results are similar to those of other studies [21]. Therefore, the parameters obtained from the PB models can be used in our following MATLAB simulation.

#### 3.2.2 Superimposed field of PB

**Fig 5:**
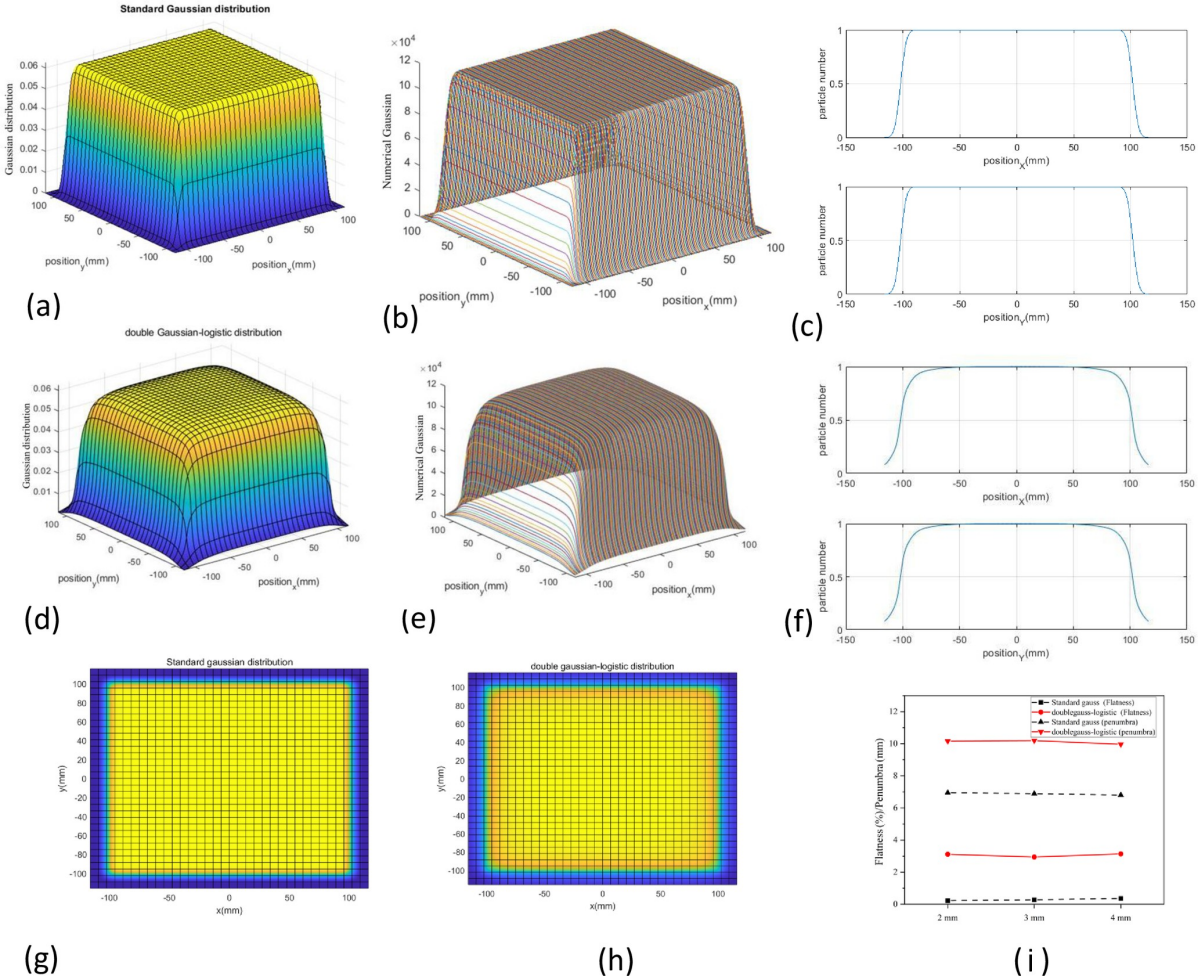
Superimposed field of PB. (a) 3D standard Gaussian distribution and (d) the double Gaussian-logistic distribution was plotted in MATLAB. (b) and (c) The projection of Gaussian distribution was drawn in both x- and y-directions. (e) and (f) The projection of double Gaussian-logistic distribution was drawn in both x- and y-directions. (g) The flat plane for standard Gaussian distribution is plotted. (h) The flat plane for double gaussian-logistic distribution is plotted. (i) The calculated flatness and penumbra were compared in the standard Gaussian model and the double Gaussian-logistic model.

To facilitate the graphical display of simulation results and reduce the commissioning time, we used MATLAB to superimpose the field of PB. The maximum treatment field is 20×20 cm^2^ at the HIMM. In clinical practice, 190 MeV/u and 260 MeV/u are universally energy. We chose 190 MeV/u as an example to study the influence of delivery uncertainty of active spot scanning on dose uniformity and penumbra in the treatment field. First, we superimposed fields of 20×20 cm^2^ using the double Gaussian model (hereafter referred to as the standard Gaussian model) and double Gaussian-logistic models for 190 MeV/u beam (Fig 5(A)–5(H)).

From the calculation results, we found that the treatment field dose distribution for the double Gaussian logistic model was smoother than that of the standard Gaussian model (Fig 5(A)–5(H)). Because we need to form a uniform transverse dose distribution, we need to determine the interval distance between each spot.

By calculating the flatness and penumbra in the x-direction, the simulations demonstrated that the flatness was within 5% based on the standard Gaussian model and double Gaussian-logistic model under ideal conditions. However, the penumbra in the double Gaussian-logistic model is about 10 mm, which is larger than the standard Gaussian model (approximately 6 mm) (Fig 5(I)). The flatness can meet the clinical requirements for both beam models at 2–4 mm spot spacing scenario. To reduce the treatment period as much as possible, we used a 4 mm spot spacing for the following study.

### 3.3 The influence of different types of errors

#### 3.3.1 The influence of different spot sizes on flatness and penumbra

**Fig 6:**
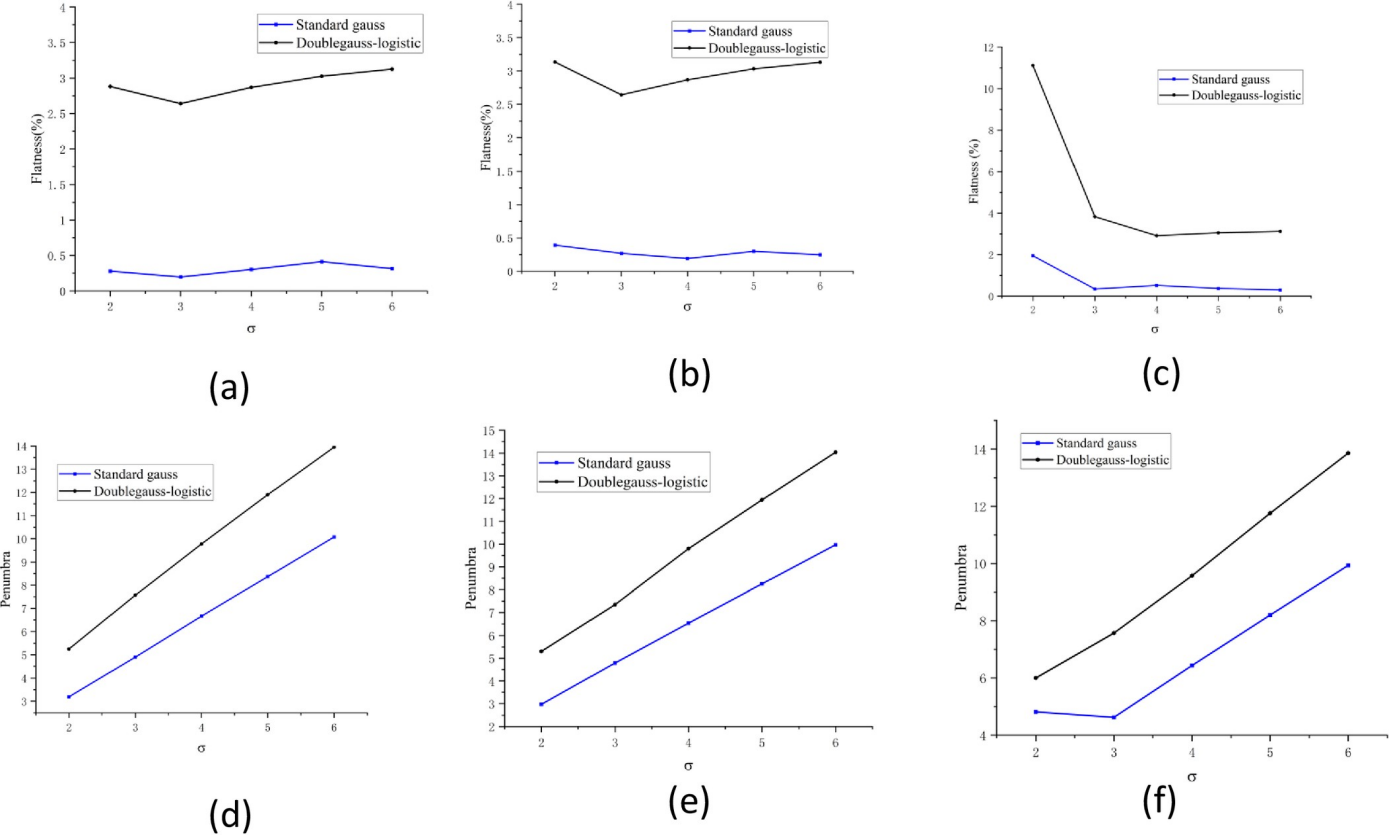
The influence of different spot sizes on flatness and penumbra. (a-c) The influence of different beam spot (2–6 mm) on dose uniformity and (d-f) penumbra for PB scanning (PBS) CIRT.

Spot size is a vital factor for PBS CIRT. The systematic error will result in a spot size change. Therefore, we calculated the flatness and penumbra at different spot sizes (σ ranging from 2 mm to 6 mm). From the overall perspective, the flatness and penumbra for the double Gaussian-logistic model is higher than that of the standard Gaussian model. The flatness is substantially smaller than 3%, which can meet the clinical requirement (except 4 mm spot spacing and 2 mm σ) (Fig 6(A)–6(C)). For heavy ion scanning beam radiotherapy, there is no error in the beam delivery system under ideal conditions, which can be obtained by using MATLAB software simulation. These results are consistent with the results of Xing *et al*. [27]. The flatness is within 3% when the quotient of the spot size (FWHM) is divided by the spot grid size larger than 2.5. As for the penumbra, the double Gaussian logistic model is also higher than the standard Gaussian model for the same spot size (Fig 6(D)–6(F)). In both beam models, with the increase in spot size, the penumbra increases accordingly. The penumbra can be constrained to 15 mm in both beam models.

#### 3.3.2 The influence of delivered dose accuracy on flatness and penumbra

**Fig 7:**
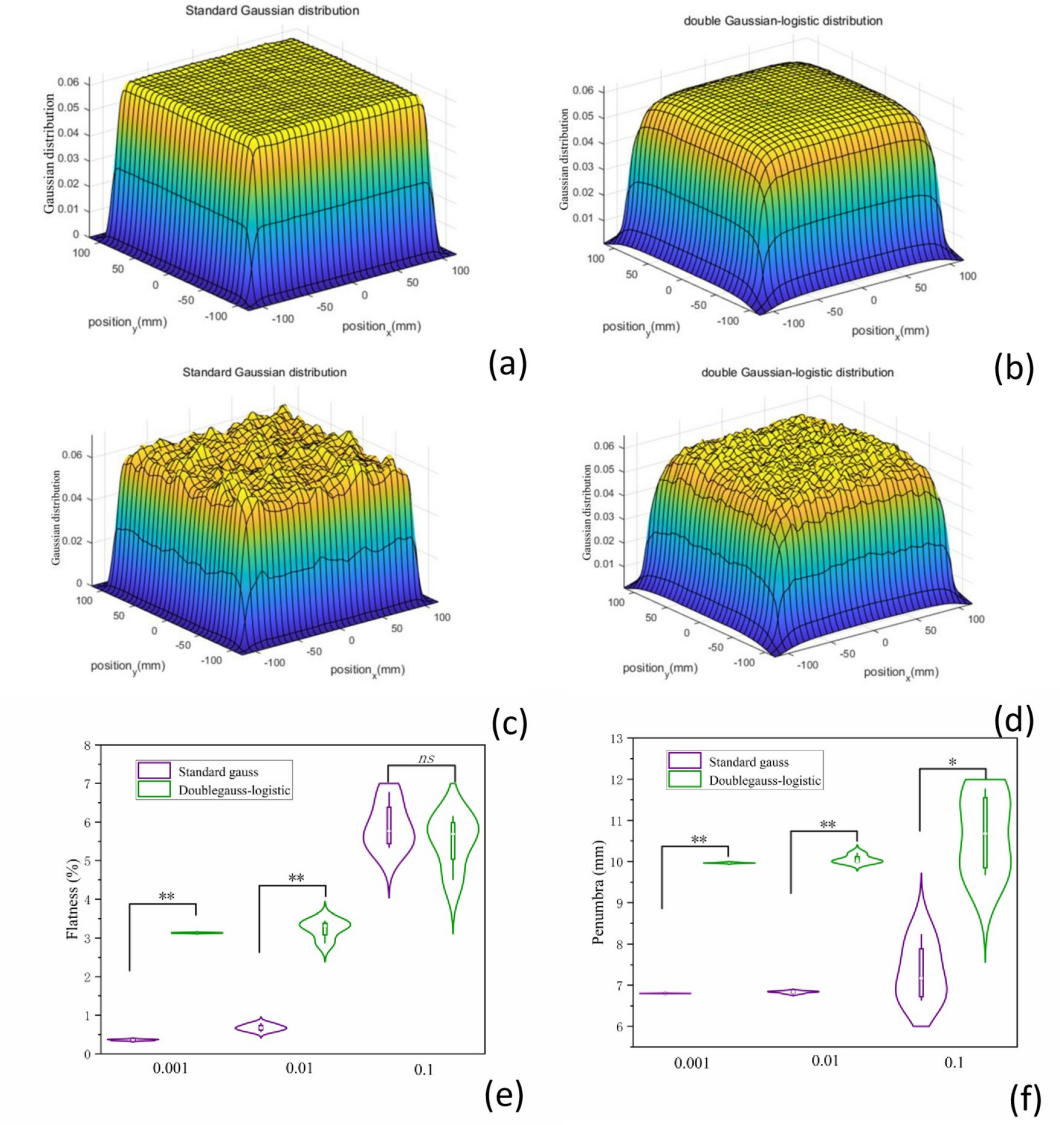
The influence of delivered dose accuracy on flatness and penumbra. (a-d) 3D Gaussian distribution with delivered dose error was plotted by MATLAB. (e) The influence of different random error (0.001, 0.01 and 0.1) on dose flatness, and (f) penumbra for PB scanning (PBS) CIRT.

To calculate the influence of spot dose accuracy on the dose flatness and penumbra, we used the *random* function to assign 0.001, 0.01, and 0.1 random errors to each spot beam (Fig 7(A)–7(D)). The calculated results show that the flatness and penumbra for the double Gaussian-logistic model is higher than the standard Gaussian model for 0.001and 0.01 (Fig 7(E) and 7(F), *P*<0.01). In addition, the penumbra for the double Gaussian-logistic model was also large when the random error increased to 0.1. However, the flatness in these two models is not significant when the random error increases to 0.1.

#### 3.3.3 The influence of random loss points on flatness and penumbra

**Fig 8:**
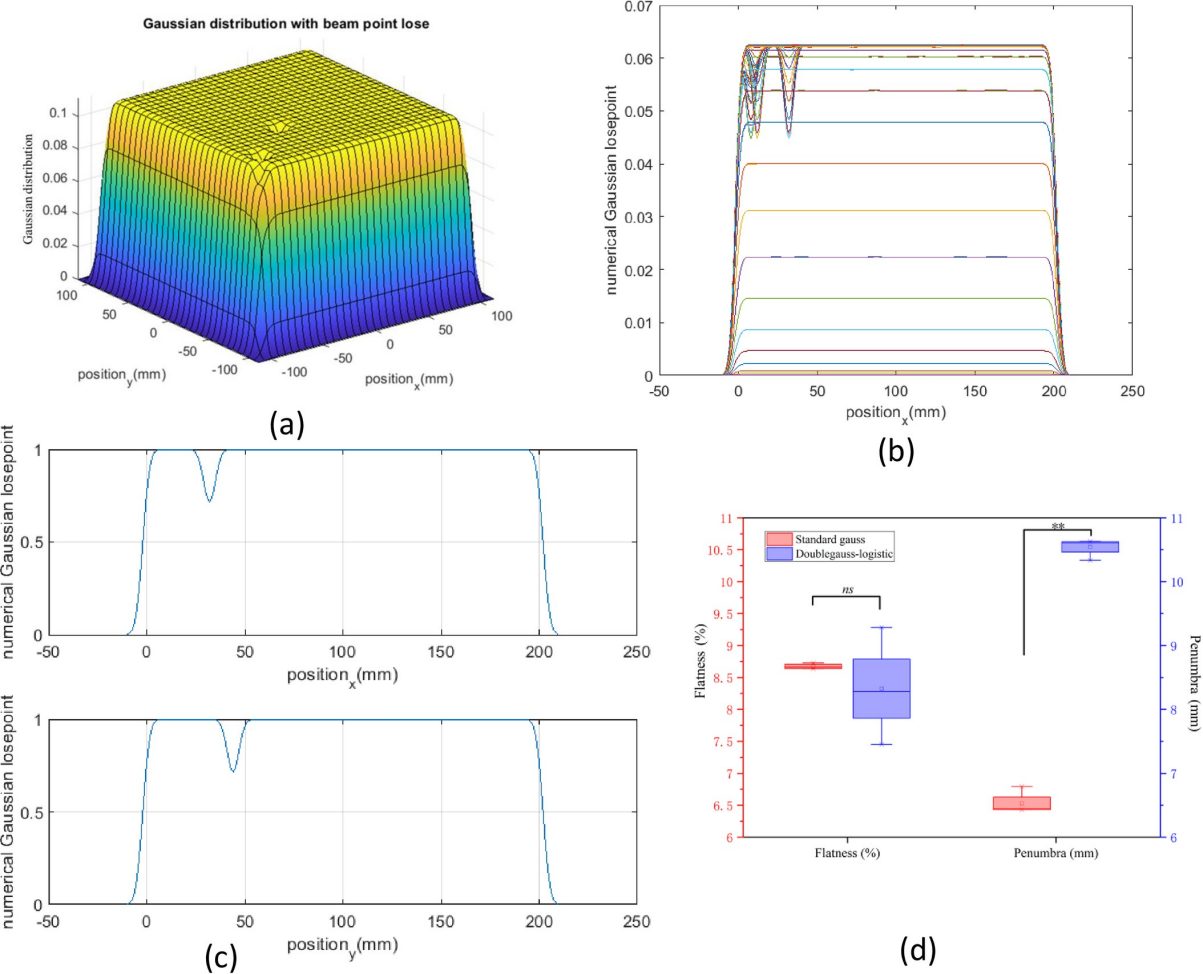
The influence of random loss points on flatness and penumbra. (a) 3D Gaussian distribution with beam point loss was plotted by MATLAB. (b) and (c) The projection of Gaussian distribution was drawn in both x- and y-directions. (d) The calculated flatness and penumbra were compared in the standard Gaussian model and the double Gaussian-logistic model. p values: **<0.01. Non-significant differences are indicated as n.s.

In the process of radiotherapy, the instability of the magnet power supply may cause the loss of a beam spot, which will affect the flatness and penumbra of the control field. Using *the find* function, the random point was lost (Fig 8(A)–8(C)). From the calculated results, we found that when the point was lost in the treatment field, the flatness was profoundly impaired. The standard Gaussian model and the double Gaussian-logistic model have similar flatness (7.75–8.75%), and there was no difference between these two models. However, as for penumbra, the double Gaussian-logistic model (10.5 mm) is significantly larger than the standard Gaussian model (6.5 mm) (Fig 8(D), *P* < 0.01).

#### 3.3.4 The influence of random loss lines on flatness and penumbra

**Fig 9:**
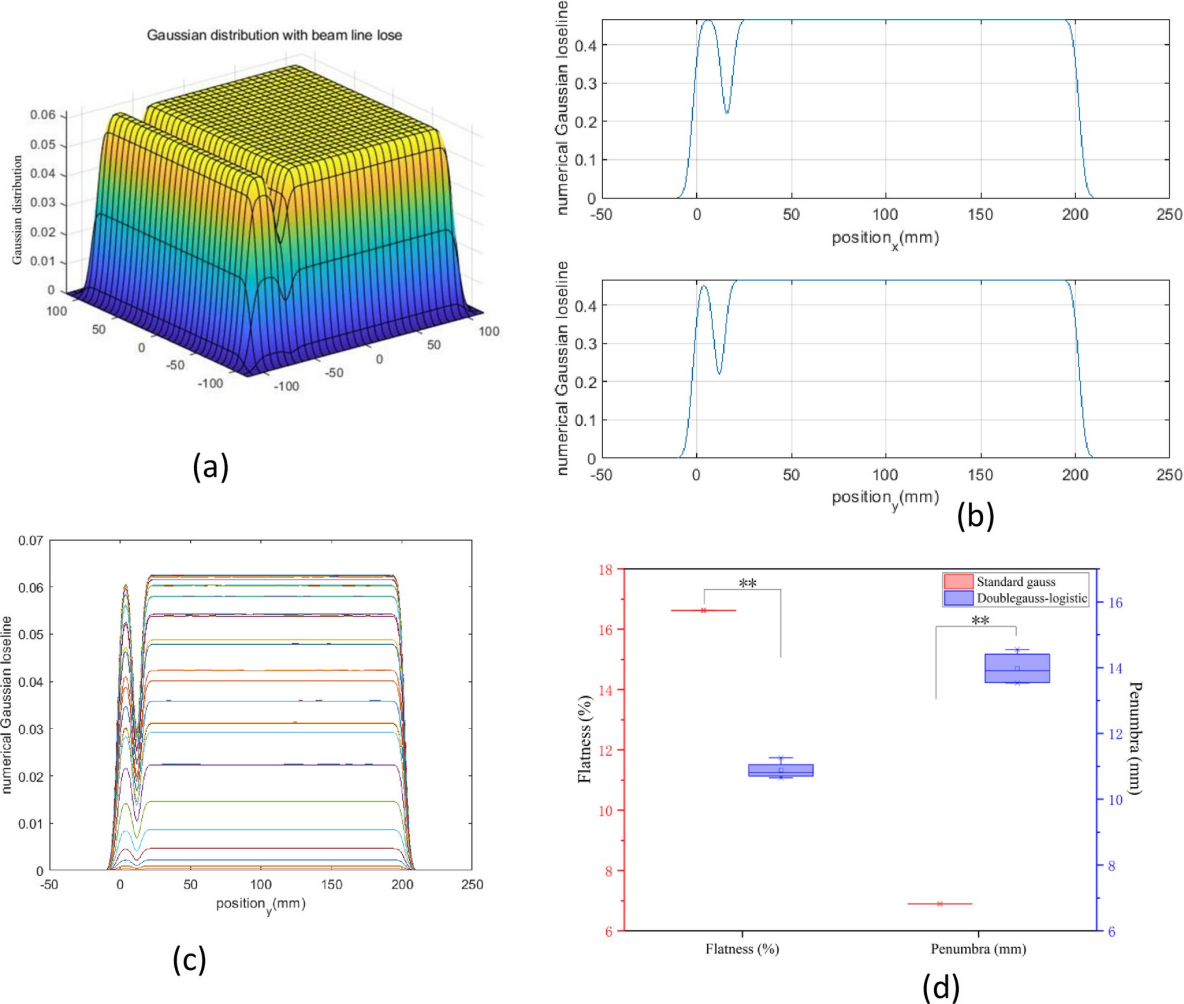
The influence of random loss lines on flatness and penumbra. (a) 3D Gaussian distribution with beam line loss was plotted by MATLAB. (b) and (c) The projection of Gaussian distribution was drawn in both x- and y-directions. (d) The calculated flatness and penumbra were compared in the standard Gaussian model and the double Gaussian-logistic model. p values: **<0.01.

Under some serious circumstances, for example, the persistent accumulation of hysteresis effect, misaligned elements, or the large positioning error of patients, may lead to the loss of a row of beam spots in the treatment field, which is a terrible uncertainty of the beam delivery system. Though using *find* and *unidrnd* function, four lines were lost at different positions (Fig 9(A)–9(C)). From the calculated results, we found that when the line was lost in the treatment field, the flatness was profoundly impaired in both beam models. The flatness in the standard Gaussian model (16.6%) was larger than that in the double Gaussian-logistic model (11.2%) (Fig 9(D), *P* < 0.01). However, as for penumbra, the double Gaussian-logistic model (13.85 mm) is significantly larger than the standard Gaussian model (6.75 mm) (*P* < 0.01).

#### 3.3.5 The influence of spot position error on flatness and penumbra

**Fig 10:**
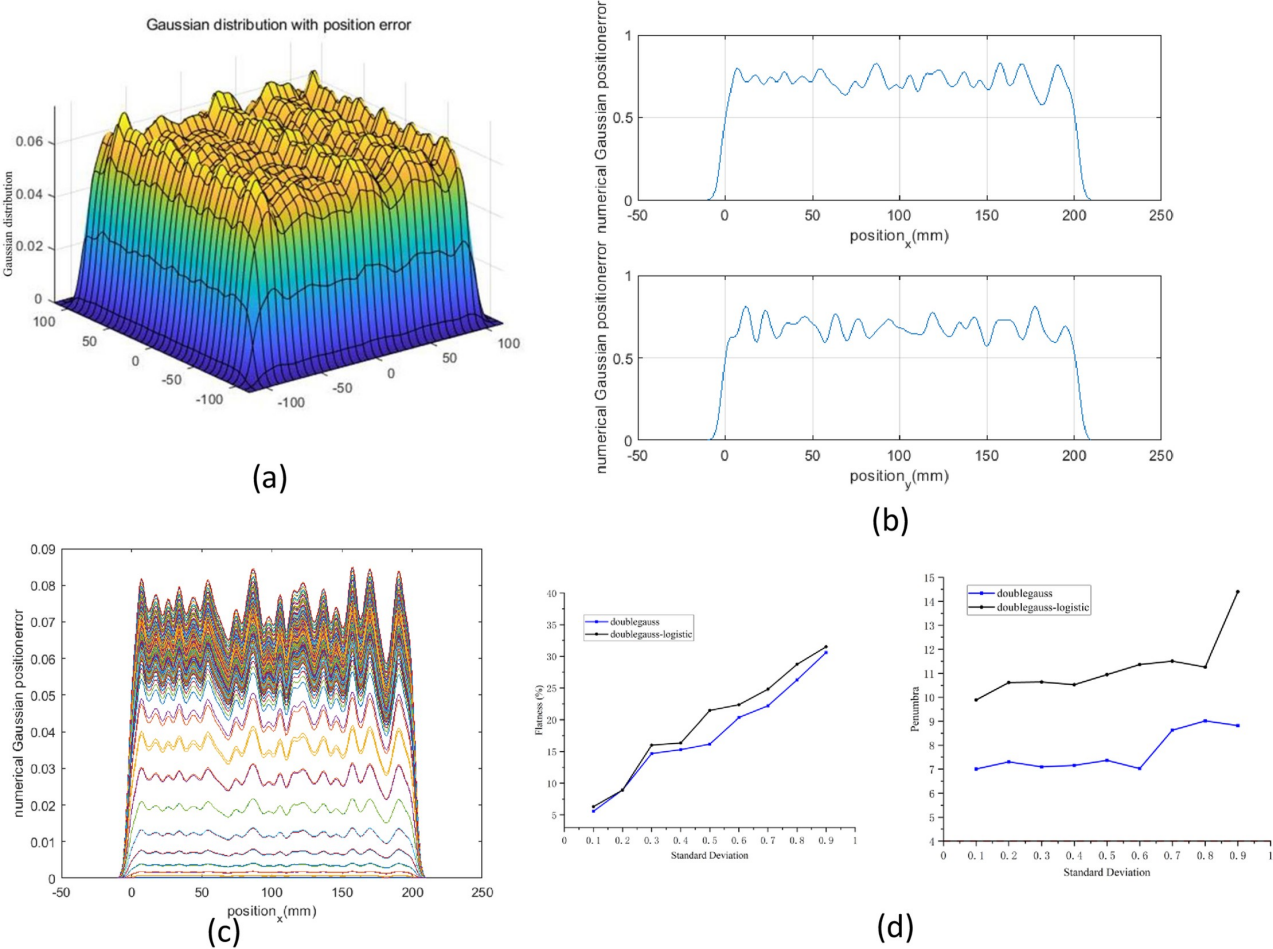
The influence of spot position error on flatness and penumbra. (a) 3D Gaussian distribution with beam spot position error was plotted by MATLAB. (b) and (c) The projection of Gaussian distribution was drawn in both x- and y-directions. (d) The calculated flatness and penumbra were compared in the standard Gaussian model and the double Gaussian-logistic model (left and right panel).

Spot position error is a common phenomenon for all radiotherapy facilities. Therefore, we used a Gaussian-like random error distribution (the standard deviation ranged from 0.1 to 0.9) to model the spot position error and compare the difference between these two beam models. A typical spot position error Gaussian distribution and projection is shown in Fig 10(A)–10(C).

From the calculated results, we found that when spot position error in the treatment field, the flatness was profoundly impaired in both beam models (larger than 5% in both beam models), that is, with an increase in the standard deviation, the flatness value is increases (Fig 10(D), left panel). The standard Gaussian model and the double Gaussian-logistic model have a similar tendency as the standard deviation of the Gaussian-like random error distribution. However, for the penumbra, the double Gaussian-logistic model (higher than 9.5 mm) is larger than the standard Gaussian model (lower than 8.5 mm) (Fig 10(D), right panel).

## 4. Discussion

In this study, we first measured the relative dose distribution, and we used MC to simulate the depth-dose distribution of 190 MeV/u and 260 MeV/u carbon-ion beams on the HIMM treatment nozzle. Although the MC simulated value is in good agreement with the experimentally measured value, there is some difference in the physical absorbed dose between the simulation and calculation of the HIMM model. The difference in measurement accuracy resulted from the finite size of the chamber, which is not large enough to capture the total scattering dose, especially for low-energy beams. In clinical practice, many different energies are necessary; we only simulated and measured two energies. Therefore, we should measure more depth-dose curves for different energies and compare them with simulation results in order to provide dose distribution input data for future investigation.

Despite the fact that PB algorithms are not optimal for accurately describing the dose deposition at interfaces of heterogeneity materials, their use for dose calculation is still widespread in clinical practice because of their faster calculation times and efficient clinical workflows. Therefore, we analyzed the three-dimensional dose distribution simulated from the MC code, and we used different PB models to fit the spatial distribution. We found that the double Gaussian-logistic model is a better beam model. The simulated profiles were finally fitted with different Gaussian distributions that did not perfectly describe the nature of the data, thus requiring a careful choice of the fitting conditions. Although the standard Gaussian models do not perfectly describe the nature of the beams, the MC-based parametrization input into the commercial TPS clearly provides an improvement over the single Gaussian approximation [19]. Upgrading the PB models by adding a description of the low-dose envelope may improve the accuracy of treatment planning [20]. However, in reality, human tissue is a heterogeneous material, so the PB we fit cannot truly reflect the spatial distribution of the human body. Therefore, we will study the spatial dose distribution in tissue-like materials in the future, such as anthropomorphic radiotherapy phantoms [32].

The PB model provides parameters for us to model a 20×20 cm^2^ treatment field. We analyzed the influence of the spot size and spot spacing, point loss, line loss, and spot position error on the dose flatness and penumbra in the treatment field based on PB algorithm models. Our study indicated that the standard Gaussian model in some situations can fulfill the lateral flatness requirement in our facility. However, as for the double Gaussian-logistic model, there is seldom a situation that fulfills the consideration of spot beam uncertainty. These results hint us that we should consider the low dose envelope in TPS algorithm. Xing *et al*. studied the homogeneity of proton and carbon ion scanning beams through a combination of different spot sizes and grid sizes based on Syngo TPS simulation and film measurements [27]. We calculated the homogeneity and penumbra of carbon ion scanning beams through a combination of different spot sizes and grid sizes. They found that the optimal ratio of spot size to spot spacing, 1.06, will fulfill the lateral homogeneity requirement in their facility. These findings are in agreement with the results of Xing *et al*. [27]. When the ratio of σ and spot spacing is larger than 1, the flatness will fulfill the clinical need (Fig 6(A)–6(C)) without considering the uncertainties. Different delivery systems may have different delivery accuracies, which may require different ratio values to achieve the same homogeneity. In addition, they only used a Gaussian-like random error distribution to model delivery uncertainties, and modeled different types of uncertainties in the beam delivery system. We found that the spot spacing decreases relative to the spot size, and the overlap of lateral profiles is greater, which contributes to the robustness of dose homogeneity [6]. However, when the spot size increases, the penumbra also increases.

The eddy current loss and hysteresis effect of the magnet and accidental power supply fluctuation will cause an error in the beam position, even resulting in loss spot and loss line in the treatment field, which results in the homogeneity and penumbra of the delivered field. In addition, misaligned elements and the patient’s setup error are also very important factors for the flatness and penumbra of the treatment field. To give full play to the advantages of a carbon‐ion therapy device, precise dosing and irradiation position monitoring as well as robust safety interlock are all necessary conditions. Through our results, we found that the calculated flatness and penumbra in the double Gaussian-logistic model is larger than that of the standard Gaussian model. Therefore, we should consider delivery uncertainty and the PB model for robust plan optimization and its possible influence on dose volume histograms in future studies. In addition, in terms of physical layout design, we can add Ta scatters or a pre-absorber to optimize the penumbra by adjusting the lateral and longitudinal sizes of scanned carbon-ion beam spots [8, 33].

MC simulation requires too much time to calculate the 3D dose distribution when the number of primary particles increases. We can consider using the MC code to generate depth dose curves and dose distribution data under different energies, and store them as a library for subsequent function calls. The MATLAB simulation can be completed in the shortest time after the relevant parameters are determined according to the MC simulation. In addition, it takes a long time to stack the grid dose in the simulation program. In a future study, GPU-based parallel computing will shorten the time needed for carrying out the simulations. In this way, not only the simulation speed is fast, but also the visual simulation results are obtained, which are worthy of reference for future investigations and future radiotherapy planning systems to be more accurate.

## Supporting information

S1 File (ZIP)
